# “To survey or to register” is that the question for estimating population incidence of injuries?

**DOI:** 10.1186/s13690-018-0322-0

**Published:** 2018-12-17

**Authors:** Dritan Bejko, Maria Ruiz-Castell, Anna Schritz, Bjarne Laursen, Rupert Kisser, Wim Rogmans, Ronan A. Lyons, Huib Valkenberg, Samantha Turner, Robert Bauer, Gabrielle Ellsaesser, Nathalie de Rekeneire

**Affiliations:** 1 Luxembourg Institute of Health, Strassen, Luxembourg; 2 National Institute of Public Health, Copenhagen, Denmark; 3 Eurosafe, Vienna, Austria; 4 Eurosafe, Amsterdam, The Netherlands; 5 Farr Institute Swansea University, Medical School, Swansea, UK; 6 Public Health Wales NHS Trust, Swansea, UK; 7 Consumer Safety Institute, Amsterdam, The Netherlands; 8 Austrian Road Safety Board, Vienna, Austria; 9 Landesamt Brandenburg für Umwelt, Gesundheit und Verbraucherschutz, Berlin, Germany; 10 Directorate of Health, Luxembourg, Luxembourg

**Keywords:** Injuries incidence, Survey, Register, Home and leisure accidents, Hospital treated injuries, BRIDGE health

## Abstract

**Background:**

Measuring the true incidence of injury or medically attended injury is challenging. Population surveys, despite problems with recall and selection bias, remain the only source of information for injury incidence calculation in many countries. Emergency department (ED) registry based data provide an alternative source.

The aim of this study is to compare the yearly incidence of hospital treated Home and Leisure Injuries (HLI), and Road Traffic Injuries (RTI) estimated by survey-based and register-based methods and combine information from both sources in to a comprehensive injury burden pyramide.

**Methods:**

Data from Luxemburg’s European Health Examination Survey (EHES-LUX), European Health Interview Survey (EHIS) and ED surveillance system Injury Data Base (IDB) collected in 2013, were used. EHES-LUX data on 1529 residents 25–64 years old, were collected between February 2013–January 2015. EHIS data on 4004 other residents aged 15+ years old, were collected between February and December 2014. Participants reported last year’s injuries at home, leisure and traffic and treatment received. Two-sided exact binomial tests were used to compare incidences from registry with the incidences of each survey by age group and prevention domain. Data from surveys and register were combined to build an RTI and HLI burden pyramide for the 25–64 years old. This project was part of the European Union project BRIDGE-Health (BRidging Information and Data Generation for Evidence-based Health Policy and Research).

**Results:**

Among 25–64 years old the incidence of hospital treated injuries per thousand population was 60.1 (95% CI: 59.2–60.9) according to IDB, 62.1 (95% CI: 50.6–75.4) according to EHES-LUX and 53.2 (95% CI: 45.0–62.4) according to EHIS. The incidence of hospital admissions was 3.7 (95% CI: 3.5–4.0) per thousand population from IDB-Luxembourg, 12.4 (95% CI: 7.5–19.3) from EHES-LUX and 18.0 (95% CI: 13.3–23.8) from EHIS. For 15+ years-old incidence of hospital treated HLI was 62.8 (95% CI: 62.1–63.5) per thousand population according to IDB whereas the corresponding EHIS estimate was lower at 46.9 (95% CI: 40.4–54.0). About half of HLI and RTI of the 25–64 years old were treated in hospital.

**Conclusion:**

The overall incidence estimate of hospital treated injuries from both methods does not differ for the 25–64 years old. Surveys overestimate the number of hospital admissions, probably due to memory bias. For people aged 15+ years, the survey estimate is lower than the register estimate for hospital treated HLI injuries, probably due to selection and recall biases. ED based registry data is to be preferred as single source for estimating the incidence of hospital treated injuries in all age groups.

## Background

Injury is the fourth leading cause of mortality in the general population and the leading cause for children above one year and adults to 44 years [1]. In order to estimate burden of injuries, set-up priorities, target groups at higher risk for prevention activities and evaluate the effects of preventive actions, decision makers need information about the incidence of both fatal and non-fatal injuries.

For injury mortality, death certificate data are commonly used, however, the method of collecting information on non-fatal injuries varies from country to country. Hospital treated injuries are best estimated using Emergency Department’s Registry (EDR) based data [2]. National Hospital Discharge Registries (HDR) are also a valuable source of information [3, 4] especially if EDR are not available from a representative sample of hospitals. Although data on specific injuries, like road traffic or work-related injuries, are collected from other organisations outside the health sector, information on the majority of out of hospital treated or untreated injuries can only be collected through surveys. In some countries surveys remain the only source of information for hospital treated injuries.

Understanding factors related to the method of data collection is crucial in accurately estimating non-fatal injury incidence and burden. Comparisons between surveys and EDR data have previously been attempted. The heterogenous approaches used pushed the authors to conclude that the results from the two methods were uncomparable [5]. One study compared a sample of cases receiving treatment for injury in a limited number of EDs, with a representative sample of the population from a survey and reported lower injury incidence in the survey [5, 6]. EDR injury data collection based only on reference trauma centers has been shown to underestimate incidence of road traffic or work-related injuries compared to other sources of data [7, 8].

Taking advantage of the small size of Luxembourg, this study provides a unique opportunity to compare national estimates of non-fatal injury incidence using data collected from all emergency departments of all hospitals in 1 year with survey based data from two representative samples of residents covering approximately the same period of time.

The aim of the present study was to compare the population incidence of hospital treated home and leisure and road traffic injuries in specific age groups estimated by survey-based and register-based methods and create an injury burden pyramid combining information from both sources.

## Methods

Cross-sectional population-based survey data from the European Health Examination Survey (EHES-LUX) and European Health Interview Survey (EHIS) in Luxembourg and data from the Luxemburg IDB system were used. For each survey a one stage random sample stratified by age group, gender and district of residence was drawn from the national health insurance register. People living in institutions such as nursing homes, hospitals or prisons were excluded. Calculating a response rate of about 25%, the number of selected individuals was 6475 residents 25–64 years old for EHES-LUX and 16,000 residents ≥15 years old for EHIS. An invitation together with an information booklet on the survey, a response form and a pre-paid envelope was sent by mail to selected individuals. Non-responders were re-contacted after a period of 3 weeks. Individuals that accepted to participate in EHIS, received an English, French, German or Portuguese questionnaire by mail or completed the web version of the questionnaire [9]. Those who agreed to participate in EHES-LUX were contacted to arrange an appointment in one of the three survey sites situated in the north, center or south of the country. After signing an informed consent, a research nurse conducted the interview in one of the four languages. The German, French and Portuguese language questionnaires used for EHIS and EHES-LUX were validated against the original English version through a translation and back translation process. Both EHES-LUX and EHIS methodology followed international guidelines and protocols [10, 11].

Out of the selected 6475 individuals 5672 were eligible and received the invitation to participate. 1902 accepted to participate and 1529 participated in EHES-LUX. The main reasons for non- participation were exclusion due to age (> 64 years old), invalid or unresolved address and negative or no reply. Data on the 1529 residents 25–64 years old, were collected between February 2013 and January 2015 [12]. Participants were asked separate questions about the previous 12 months injuries at home, during leisure activities, at work, about Road Traffic Injuries (RTI) while commuting to work and non-work related RTIs. From respondents declaring one or more injuries, information was collected on treatment received for each injury with the following answer options: admitted and stayed overnight in hospital; admitted but did not stay overnight in hospital; treated by a doctor or nurse outside the hospital; and no consultation or intervention was necessary.

For EHIS, 4823 out of the selected 16,000 individuals accepted to participate, 4118 satisfied the inclusion criteria, signed the informed consent and completed the questionnaire. For 4004 questionaires the completition rate was above 50% and there were no missing data on age, sex and district of residence [9]. EHIS data on 4004 residents ≥15 years old, of whom 2794 aged 25–65 years old, were collected between February and December 2014. Participants were asked in three separate questions if they had experienced injuries at home, during leisure activities or from road traffic during the previous year [11]. Only for the most severe injury the information on received treatment was collected using the same answer options as for EHES-LUX.

Age, sex and district of residence was used to check for differences between responders and non-responders in both surveys. For EHES-LUX there was an overrepresentation of females, individuals from the east region of the country and individuals aged 45–54 years old among responders. For EHIS there was no difference between responders and non responders according to the disctrict of residence. However, an overrepresentation of females and an underrepresentation of individuals older than 85 years or younger than 25 years was found among responders. To make the responders representative of Luxembourg’s population in terms of age, sex and area of residence, sampling weights were calculated from the selection probabilities, using Luxembourg census data in 2011 as a reference and were adjusted for non-response [13]. 140 individuals did not answer injury questions in EHIS. Therefore, sensitivity analysis was performed by once including all non-responders as if they experienced a hospital treated injury and once including all non-responders as if they were not injured during the previous year. In EHES-LUX, as only one individual did not answer questions about injuries, no sensitivity analysis taking into account the non-response was performed.

Registry data from the Luxembourg ED injury surveillance system in 2013 was used for comparison. The common European Injury Data Base (IDB) methodology is used by IDB-Luxembourg [14]. Injury cases are selected based on the reason of visit registered by a nurse at ED’s triage or if at least one International Classification of Diseases (ICD-10) code of injury is used by the medical doctor. In hospitals that were using a paper and pencil system, files of all ED patients were reviewed, injury cases were selected and coded by a data entry clerk. Monitoring visits on randomly selected weekdays and weekends were performed in all EDs to check for completeness according to the World’s Health Organization methodology [15]. Finally, narratives extracted together with injury data were reviewed, to exclude non-cases and validate the data. After a pilot phase launched in 2012, all nine EDs regrouped in five hospitals in Luxembourg participated in IDB-Luxembourg in 2013.

As per IDB-Network methodology a detailed set of information called the Full Data Set (FDS) is collected in one hospital. All other hospitals collect less detailed information corresponding to the IDB- Minimum Data Set (MDS). Only first visit for an injury was considered as a case and non-residents were excluded from the calculations. Both FDS and MDS include items, such as intent (accident, self-harm or violence), activity (sport, paid work), place of occurrence (home, school or road) and mechanism (fall, burn, road traffic injury, etc.). Combining information from different fields makes it possible to classify injuries according to prevention domains. Road Traffic Injuries are all injuries for which the mechanism is a road traffic accident, including those while commuting to work. The home and leisure injury group includes all unintentional injuries excluding those from road traffic, occupational exposures and occurring in schools [16]. Given the definition of leisure time injuries in the surveys, injuries classified as due to sports from IDB were also included in the home and leisure category.

For comparative reasons with EHIS, work and non-work related road traffic injuries from EHES-LUX were regrouped into the RTI group. Only the most serious medical care intervention for the most serious injury event was considered for incidence calculation in EHES-LUX. Home and leisure time accidents were combined into Home and Leisure injuries (HLI). Information collected in the surveys aboutthe proportion of injury cases receiving out of hospital treatment or not receiving any medical treatment were used to build the injury pyramide. The total number of injuries was calculated by dividing the number of hospital treated HLI and RTI from IDB with the proportion of hospital treated HLI and RTI estimated by EHIS and EHES-LUX.

For IDB-Luxembourg, incidence was calculated by dividing the number of cases registered in IDB-Luxembourg for the specific age groups and prevention domain by the total number of residents of that age group, as recorded in the official statistics of 2013 [17]. For surveys, incidence was calculated by dividing the number of participants reporting receiving medical treatment in hospitals for a specific age-group and prevention domain by the total number of participants of the same age-group. Non-responses to questions about the previous year’s injuries were excluded from calculation. For the surveys and the registry, 95% confidence intervals (95% CI) were calculated. The annual incidence estimates from the IDB registry were considered as true population data for 2013. Two-sided exact binomial test was used to compare incidences of IDB versus EHES-LUX, and IDB versus EHIS. For surveys estimates, both weighted and unweighted data were presented but conclusions were based only on the weighted estimates.

EHES-LUX and IDB-Luxembourg had received prior ethical approval from Luxembourg’s National Ethic’s Comitte, the Comite National d’Ethique de Recherche (CNER). According to national regulation and responding to the european obligation to collect EHIS data, the CNER was informed by Luxembourg’s Ministry of Health in charge of EHIS. All survey participants signed a prior informed consent. Only anonymous unlinkable data were included in IDB-Luxembourg and in EHIS. Information about EHES-LUX, EHIS and IDB-Luxembourg was sent to the Nationnal Data Protection Comittte prior to data collection. This work comprised part of the methodological development work on injury surveillance for the EU funded BRIDGE-Health (BRidging Information and Data Generation for Evidence-based Health Policy and Research) project.

## Results

**Fig. 1:**
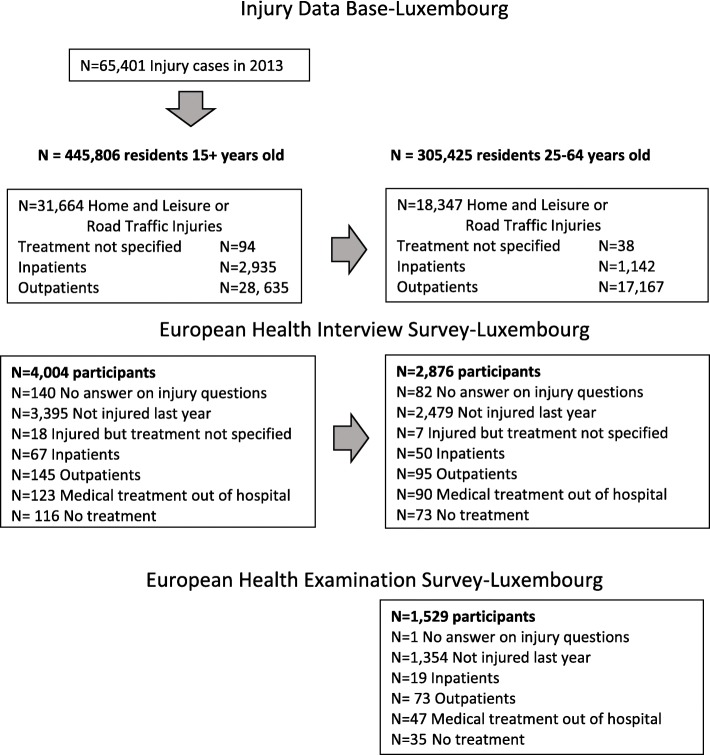
Flow chart with inclusion of injury cases for IDB Luxembourg, EHIS and EHES-LUX surveys

A total of 65,401 injury cases were registered in IDB-Luxembourg in 2013. There were 18,347 residents aged 25–64 years old that received medical care in one of the hospitals of the country because of a HLI or RTI. Among them 1142 (6.2%) were hospitalised (Fig. 1). The total number of HLI and RTI among the ≥15 year old residents was 31,664 and among these 2935 (9.3%) were hospitalised.

Among 3864 EHIS participants aged 15+ years that responded to the injury questions, 469 reported an injury last year of whom 67 were admitted and stayed overnight, 145 were treated in hospital’s ED as outpatients, and 123 received medical care outside hospital. Focusing on 25–64 years old the corresponding figures were 315 injuries reported, 50 inpatients and 95 outpatients from 2794 respondents. Finally, among 1528 EHES-LUX participants that responded to injury questions, 174 had at least one injury last year, 19 were hospitalised and 73 were treated as outpatients.

The incidence of hospital treated road traffic injuries among 25–64 years old was 8.0 per 1000 (‰) population (95% CI: 7.7–8.3) according to IDB, 8.5‰ (95% CI: 4.5–14.5) according to EHES-LUX and 8.6‰ (95% CI: 5.5–12.9) according to EHIS (Table 1). Among 15+ years the corresponding figure were 8.3 ‰ (95% CI: 8.0–8.5) for IDB and 8.8 ‰ (95% CI: 6.1–12.3) according to EHIS.

**Table 1:** Incidence of injuries per 1000 population (‰) per age group and prevention domain according to the different methods

Injury Data Base (IDB)-Luxembourg	15–24	25–34	35–44	45–54	55–64	25–64	CI 95%		65–74	75 +	15 +	CI 95%	
Road Traffic Injuries (RTI) (n)	1029	903	702	564	270	2439			117	94	3679		
Home and Leisure injuries (HLI) (n)	5735	4778	4326	4048	2756	15,908			2056	4286	27,985		
Population at risk	65,324	78,671	84,061	82,761	59,932	305,425			39,365	35,692	445,806		
Incidence RTI (‰)	15.8	11.5	8.4	6.8	4.5	8	7.7–8.3		3.0	2.6	8.3	8.0–8.5	
Incidence HLI (‰)	87.8	60.7	51.5	48.9	46	52.1	51.3–52.9		52.2	120.1	62.8	62.1–63.5	
Incidence RTI and HLI (‰)	103.5	72.2	59.8	55.7	50.5	60.1	59.2–60.9		55.2	122.7	71.0	70.2–71.8	
European Health Examination Survey (EHES)								IDB vs EHES *p*-value					
RTI (n)		4	5	2	2	13							
HLI(n)		18	29	16	16	79							
Population at risk		314	461	461	292	1528							
Incidence RTI (‰)		12.7	10.8	4.3	6.8	8.5	4.5–14.5	0.77					
Incidence HLI (‰)		57.3	62.9	34.7	54.8	51.7	41.1–64.0	1					
HLI + RTI (‰)		70.1	73.8	39.0	61.6	60.2	48.8–73.3	0.96					
Weighted													
Incidence RTI (‰)		12.9	9.2	4.9	6.7	8.5	4.5–14.5	0.77					
Incidence HLI (‰)		62.0	64.7	34.0	53.9	53.6	42.9–66.1	0.79					
Incidence RTI and HLI (‰)		74.9	74.1	38.8	60.6	62.1	50.6–75.4	0.74					
European Health Interview Survey (EHIS)								IDB vs EHIS *p*-value					IDB vs EHIS *p*-value
RTI (n)	6	4	9	7	3	23			3	1	33		
HLI (n)	35	27	35	25	33	120			11	8	174		
RTI or HLI (n)	1	0	2	0	0	2			1	1	5		
Population at risk	461	672	723	786	613	2794			383	226	3864		
Incidence RTI (‰)	13.0	6	12.4	8.9	4.9	8.2	5.2–12.3	0.83	7.8	4.4	8.5	5.9–12.0	0.79
Incidence HLI (‰)	75.9	40.2	48.4	31.8	53.8	42.9	35.7–51.1	0.03	28.7	35.4	45.0	38.7–52.1	< 0.001
Incidence RTI and HLI (‰)	91.1	46.1	63.6	40.7	58.7	51.9	44.0–60.8	0.07	39.2	44.2	54.9	47.9–62.5	< 0.001
Weighted													
Incidence RTI (‰)	12.2	5.8	14.5	8.3	4	8.6	5.5–12.9	0.70	7.7	4.3	8.8	6.1–12.3	0.68
Incidence HLI (‰)	72.9	42.2	50.2	31.9	53.8	43.9	36.4–52.3	0.054	33.4	39.0	46.9	40.4–54.0	< 0.001
Incidence RTI and HLI (‰)	88.5	48	67.4	40.3	57.8	53.2	45.0–62.4	0.13	43.7	47.6	57.2	50.1–65.0	< 0.001

The incidence of hospital treated Home and Leisure injuries among 25–64 years old was 52.1‰ (95% CI: 51.3–52.9) according to IDB, 53.6‰ (95% CI: 42.9–66.1) according to EHES-LUX and 43.9‰ (95% CI: 36.4–52.3) according to EHIS (Table 1). Among 15+ years the corresponding figures were 62.8 ‰ (95% CI: 62.1–63.5) according to IDB and 46.9‰ (95% CI: 40.4–54.0) according to EHIS. There was no statistically significant difference between each survey and IDB-Luxembourg in estimating the incidence of hospital treated RTI or HLI among 25–64 years old.

**Fig. 2:**
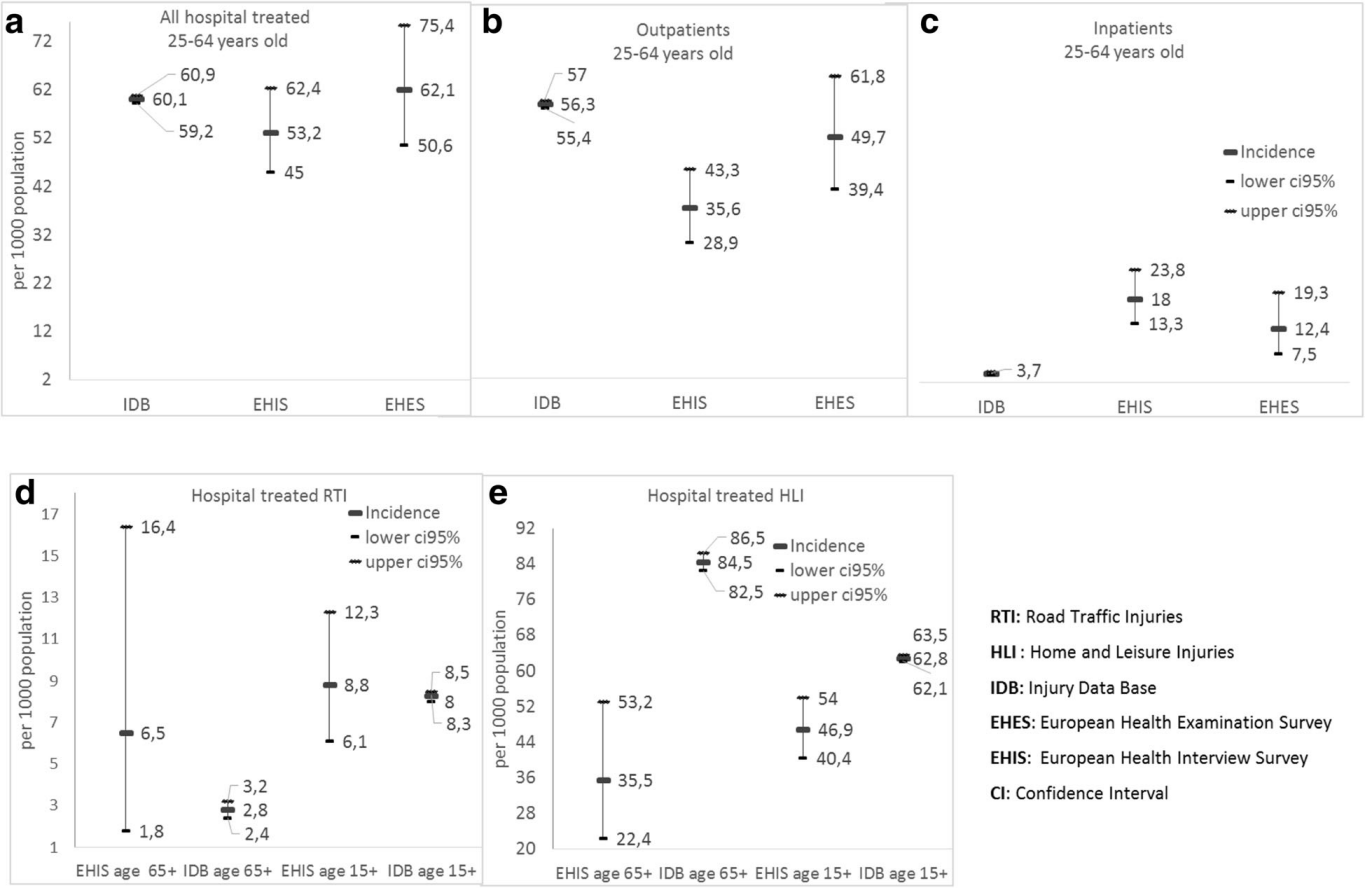
Incidence (per 1000 population) of hospital treated injuries according to method of estimation and age-group

The incidence of hospital admissions for RTI was 0.8‰ (95 %CI: 0.7–0.9) from IDB, 1.3‰ (95% CI; 0.2–4.7) from EHES-LUX and 3.7‰ (95% CI; 1.8–6.9) from EHIS (Fig. 2). The incidence of hospital admissions due to Home and Leisure injuries was 2.9‰ (95% CI; 2.8–3.1) from IDB, 11.1‰ (95% CI: 6.5–17.7) from EHES-LUX and 13.9‰ (95% CI; 9.8–19.1) from EHIS (Table 2). The incidence of hospital admisions for HLI was overestimated by both surveys and EHIS overestimated also hospital admisions for RTI (*p* < 0.001). It should be noted that for the age-group 25–64 years old, EHIS overestimated the incidence of hospital admissions, underestimated the incidence of hospital outpatients but when it comes to overall hospital treated injuries the difference between the EHIS estimate and IDB estimate was not statistically signficant (Fig. 2).

**Table 2:** Incidence of injuries per 1000 population (‰), among the 25–64 years old, per prevention domain and treatment according to the different methods

	Injury Data Base (IDB)-Luxembourg			European Health Examination Survey (EHES)				European Health Interview Survey (EHIS)			
	n	Incidence ‰	(CI95%) ‰	n	Incidence	(CI95%) ‰	IDB vs EHES *p*-value*	n	Incidence ‰	(CI95%) ‰	IDB vs EHIS *p*-value*
Hospital inpatients											
Unweighted											
Road Traffic Injuries (RTI)	242	0.8	0.7–0.9	2	1.3	0.2–4.7	0.34	9	3.2	1.5–6.1	< 0.001
Home and Leisure Injuries (HLI)	900	2.9	2.8–3.1	17	11.1	6.5–17.8	< 0.001	40	14.3	10.2–19.4	< 0.001
RTI and HLI	1142	3.7	3.5–4.0	19	12.4	7.5–19.4	< 0.001	50	17.9	13.3–23.5	< 0.001
Weighted											
RTI					1.3	0.2–4.7	0.34		3.7	1.8–6.9	< 0.001
HLI					11.1	6.5–17.7	< 0.001		13.9	9.8–19.1	< 0.001
RTI and HLI					12.4	7.5–19.3	< 0.001		18.0	13.3–23.8	< 0.001
Hospital outpatients											
Unweighted											
RTI	2191	7.2	6.9–7.5	11	7.2	3.6–12.8	0.88	14	5.0	2.7–8.4	0.21
HLI	14,976	49	48.0–50.0	62	40.6	31.2–51.7	0.14	80	28.6	22.8–35.5	< 0.001
RTI and HLI	17,167	56.3	55.4–57.0	73	47.8	37.6–59.7	0.16	95	34.0	27.6–41.4	< 0.001
Weighted											
RTI		7.2			7.2	3.6–12.8	0.88		5.2	2.9–8.8	0.22
HLI		49			42.5	33.0–53.9	0.24		30.0	23.8–37.2	< 0.001
RTI and HLI		56.3			49.7	39.4–61.8	0.27		35.6	28.9–43.3	< 0.001

*Exact Binomial Test

**Fig. 3:**
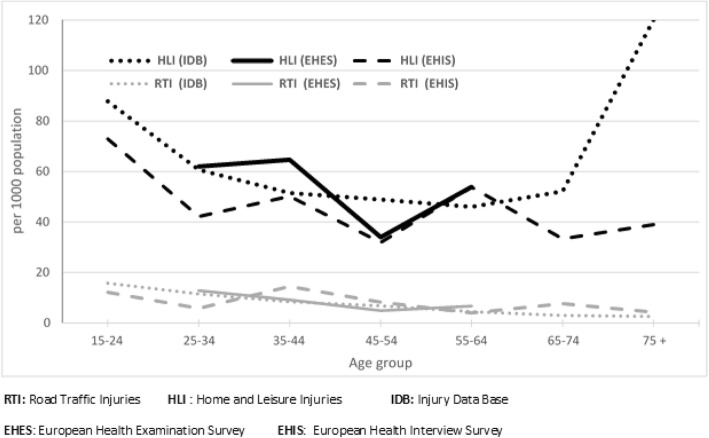
Incidence (per 1000 population) of hospitals treated HLI and RTI according to IDB-Luxembourg, EHES and EHIS surveys

According to IDB data, the incidence of hospital treated HLI injuries showed two peaks (Fig. 3), one among the 15–24 years old with 87.8 (95% CI: 85.6–90.0) per 1000 population and another among those 75+ years with 120.1 (95% CI: 116.7–123.5) per 1000. EHIS estimated an incidence of HLI among 15–24 years old of 72.9 ‰ (95% CI: 53.1–97.3) that was not different from IDB (*p*-value =0.09). For people aged 65 years or older EHIS underestimated the incidence of hospital treated HLI (35.5 vs 84.5; *p*-value < 0.001). The difference was more accentuated among the 75+ years old with an estimate of 39.0 per 1000 population, which was about three folds lower than the 120.1 per 1000 population IDB estimate (*p*-value < 0.001). On the other hand there was no difference in estimating the incidence of RTI between EHIS and IDB in any of the above mentioned age groups (Table 1).

**Fig. 4:**
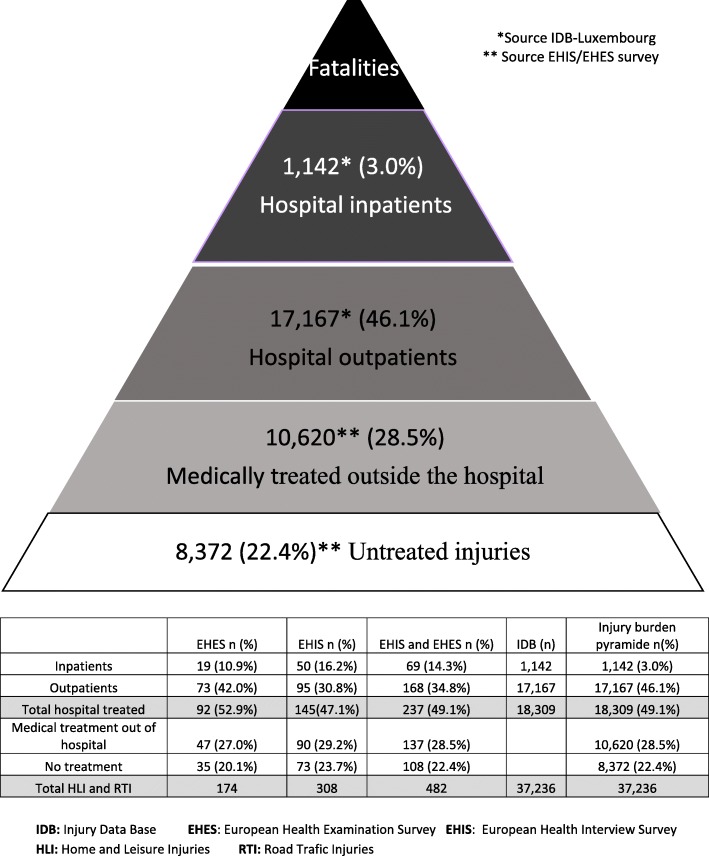
Injury pyramide for Home and Leisure injuries and road traffic accidents among 25–64 years old residents in Luxembourg in 2013

For the age group 25–64 years old we can estimate from the surveys that for HLI and RTI about 49.1% of injured cases will receive a medical treatment in a hospital, 28.4% will be treated outside the hospital and 22.4% will not be treated at all. This information was combined with absolute numbers registerd in IDB. Consequently 49.1% of all hospital treated RTI and HLI in 2013 corresponded to 18,374 registered cases. The total number of injuries was estimated to be 37,422. Finally, the share of inpatients and outpatients among hospital treated injuries provided from IDB data completes the information for an injury burden pyramid to be constructed for 2013 (Fig. 4). However, an injury burden pyramid will not be valid for the other age groups given that survey and register estimates are different.

For the sensitivity analysis of EHIS data (15+ years old), including all non-responders (*n* = 140) as individuals with hospital treated injury, the incidence of hospital treated injuries was 90.4 (95% CI: 81.7–99.7) per 1000 population, which is higher compared to the IDB incidence rate (90.4 vs. 71.0; *p*-value: < 0.001). On the other extreme, when including all non-responders as individuals without any injuries during the last year, the incidence of hospital treated injuries was 55.2 (95% CI: 48.3–62.7) per 1000 population, which is lower compared to the IDB indidence rate (55.2 vs. 71.0; *p*-value: < 0.001).

## Discussion

For people aged between 25 and 64 years the overall incidence estimate of hospital treated injuries from surveys and ED based registries was similar but the incidence of hospital admissions was overestimated by both surveys. EDR based data showed an increase in HLI incidence from age 65 which was more emphasized from 75+ years. This increase was not observed in EHIS survey data that underestimated the incidence of hospital treated HLI for this age group. For all participants > 15 years old, the incidence of hospital treated HLI was underestimated by the EHIS survey (46.9‰ population, 95% CI: 40.4–54.0) when compared to register based estimates (62.8 ‰; 95% CI: 62.1–63.5). As concluded in other studies [18–20] with only about half of all injuries treated in hospitals, the combination of both data sources provided better estimates of injury incidence but was limited in scope to selected age groups and types of injury.

Overreporting of hospitalisations from survey is also reported from other studies and is due to a specific subgroup of memory bias called telescoping bias [21]. Events like being hospitalised for an injury are brought forward in time by the responder although they have happened more than a year ago. In a Danish study data from National Health Interview Survey, participants were linked at individual level with Hospital EDR and HDR data [20]. For some cases no EDR evidence of hospital treated injuries among survey participants declaring they had an injury was found. On the other hand EDR evidence of injury among survey participants declaring they had not sustained an injury was reported. Overall, due to a combination of telescoping and recall bias no difference between surveys and ED based registries in estimating hospital treated injury incidence and an overestimation of hospital admissions by the survey was found.

Many studies report about difficulties that elderly people have to recall falls, especially for a 12 months recall period although falls causing injuries are less likely to be forgotten [22, 23]. It should be noted that in 2013 about 80% of injuries registered in IDB-Luxembourg among + 70 years old were due to falls [1].

Selection bias is not excluded given that for people older than 65 years the EHIS participants were not representative of the reference population. As a matter of fact, people from residential institutions, like nursing homes, homes for elderly, were excluded from the EHIS sampling frame. This would have a limited effect for the age group 65–75 years old given that more than 95% of people in this age-category live in their private homes. However the proportion of people living in homes for elderly increases with age from 10% for the 80 years old to 40% for the 90 years old [24]. Although after weighting, responders were representative of Luxembourg’s population on sex, age, and district of residence, a participation rate of 25% for the surveys might also be a source of selection bias. Underestimation by surveys of injuries among 65+ years old has been reported elsewhere as well [6]. By using questionnaires in different languages completed by research nurses in a face-to-face interview, EHES-LUX could accomodate disabled indindividuals. However, there was one exclusion due to handicap. For EHIS, it is likely that people with cognitive impairement or visual problems were unable to complete the questionnaire. On the other hand, IDB-Luxembourg in 2013 included all ED of all hospitals avoiding thus a selection bias reported in injury registries that collect data only in specialised trauma centers [7].

Sensitivity analyses for non-responders of injury questions in EHIS (*n* = 140) were performed by comparing the extreme cases, i.e. that either all non-responders experienced a hospital treated injury during the previous year or that all non-responders did not experience an injury. As the incidence rate of hospital treated injuries is small, the number of non-responders can influence the estimated incidence rate of the survey data. When comparing the incidence rates of the sensitivity analysis with the IDB rates, the incidence rate of the survey was overestimated when all non-responders were treated as injury cases.

Finally EHIS participants only had the possibility to report one hospital treated injury per year whereas in IDB all hospital treated injures of one particular person were counted as separate injury cases. The anonymous unlinkabel nature of IDB-Luxembourg data does not allow to see if one person has more than one hospital treated injury per year. This might have an effect on underestimating incidence from the surveys. It is reported that about 11% of nursing home residents 70+ years report more than one fall for the previous year [25].

Although cross-sectional surveys provide information on potential risk factors, there are inherent limitations in survey data for obtaining a deeper insight in determinants of injuries and their consequences. Most surveys exclude institutionalised persons and children or gather information from proxies, undermining reliability of data collected. Depending on the recall period, injury incidence will be underestimated due to the recall bias. Lack of clarity of definitions used in questionnaires might lead to measurment errors. Limited space for detailed questions will reduce the quality of information on causes and circumstances of injuries [26]. For this purpose, ED based injury surveillance systems, which allow to collect detailed information in a cost effective way on a large number of cases, are indispensable.

## Conclusions

In absence of ED based injury surveillance systems covering a representative sample of hospitals, for people aged 25–64 years old, surveys provide a valid estimate of hospital treated HLI and RTI but overestimate the the number of hospital admissions. Incidence of hospital treated HLI among people aged 65+ years and for all the age group of 15+ years old will be underestimated by surveys. With only about half of injuries receiving medical care in hospitals, combining both methods gives a better estimate of injury burden although it is limited to selected age groups and types of injury.

## Abbreviations

BRIDGE-Health: BRidging Information and Data Generation for Evidence-based Health Policy and Research
CI: Confidence interval
ED: Emergency department
EDR: Emergency Department’s Registries
EHES: European Health Examination Survey
EHIS: European Health Interview Survey
FDS: Full Data Set
HDR: Hospital Discharge Registries
HLI: Home and Leisure injuries
IDB: Injury Data Base
MDS: Minimum Data Set
RTI: Road Traffic Injuries
